# 273 Measles Post-exposure Investigations at a New York City Safety-net Hospital

**DOI:** 10.1017/ash.2026.10635

**Published:** 2026-06-23

**Authors:** Janelle Franklin, Lusungu Ngailo, Atuganile Musyani, Irene Rabiel, Mwanakombo Khama, Rita Mutayoba, Frida Ngalesoni, Anthony Ndjovu, Stella Kassone, Juhudi Mfaume, Tumaini Masegese, Ndekya Oriyo, Eninka Mmbaga, Kokuhabwa Mukurasi, Biesse D. Soura, Marcelina Mponela, Ruth Ngowi, Esther Kondo, Ally Hussein, Nsiande Lema, Mtumwa Bakari, Elizabeth Shayo, Sylvester Nandi, Pius Kagoma, Joseph Hokororo, Rose Kibirigi

**Affiliations:** 1 Centers for Disease Control and Prevention; 2 Amref Health Africa in Tanzania; 3 Amref Health Africa; 4 Amref Heath Africa; 5 Amref Health africa in Tanzania; 6 Amref Health Africa Tanzania; 7 CDC; 8 NIMR DAR-ES-SALAAM; 9 National Foundation for Centres for Disease Control; 10 Presidents Office-Regional Administration and Local Government

## Abstract

**Background:** Effective implementation of infection prevention and control (IPC) reduces the risk of healthcare-associated infections (HAIs), thereby protecting patients and healthcare workers. Tanzania has experienced ongoing outbreaks demonstrating a need for stronger IPC. Amref Health Tanzania, in collaboration with the Ministry of Health and U.S. Centers for Disease Control and Prevention, implemented a multimodal project in Kigoma, a high HAI-risk region, aimed at improving IPC practices in 20 healthcare facilities (HCF). It involved activating Quality Improvement Teams (QIT), supportive supervisions and mentorships, quarterly standardized assessments using the national Standard Based Management and Recognition (SBMR) tool, and virtual and in-person trainings. We evaluated progress and effectiveness of the project. **Methods:** We conducted a mixed methods evaluation of the project in March 2024. Ten HCFs were selected purposively to have variation on location, IPC performance, and level and convenience sampling was used to select staff. A comparative cross-sectional design was utilized to collect data through focus group discussions (FGDs), key informant interviews (KIIs), and quantitative knowledge assessments and practice observations. A comparison of SBMR results at baseline (September 2021) and endline (October 2023) was completed to assess effectiveness of the project. Quantitative data were analyzed using STATA 14 software and thematic analysis was conducted for qualitative data. **Results:** Ten FGDs, 8 KIIs, 150 knowledge assessments, and 6-11 observations per HCF were conducted. The SBMR baseline to endline comparison showed IPC performance increased for 95% of HCFs, from 49.9% to 70.7%, on average. During knowledge assessments participants verbally described accurate knowledge of IPC practices 28.0% of the time, with a higher proportion of participants from rural districts (40.8%) and medical doctors (55.6%) describing practices accurately. Based on observations, adherence to injection safety and high-level disinfection was high; weaknesses were observed for hand hygiene, waste management and spillage cleaning. Thematic analysis of qualitative data revealed strengths in the frequency of supervisions and SBMR assessments. Remaining challenges include insufficient infrastructure, supplies, and HCW IPC knowledge on implementation of IPC practices, such as hand hygiene. **Conclusion:** Although the comparison of baseline and endline SBMR results show progress, IPC knowledge and adherence to evidence-based practices remain low three years into the IPC project, further hindered by insufficient infrastructure and resources. Addressing the root cause of the identified gaps, such as ensuring sufficient IPC supplies and practical training, is critical for sustained IPC progress. These results will be used to improve the delivery of this multimodal IPC project.

